# Delaying Tackling in Youth Contact Sports: Moving toward a Safer Future or Softening the Game?

**DOI:** 10.1007/s40279-025-02236-z

**Published:** 2025-05-03

**Authors:** Joel M. Garrett, Jonathon Headrick

**Affiliations:** School of Health Sciences and Social Work, Griffith University, 1 Parklands Dr, Southport, QLD 4215 Australia

## Abstract

This current opinion article evaluates the rationale for delaying or modifying tackling in youth contact sports until age 12 years. There is growing concern that young athletes may be at heightened risk of sports-related concussions (SRC) and other injuries due to their developing brains and less-developed neuromuscular control. Recent studies exploring non-tackle formats, weight categories and body-checking restrictions demonstrate the potential benefits of reducing high-impact collisions at younger ages without the detrimental effects, i.e., increased SRC rate, when contact is introduced. Yet, critics may worry that delayed contact, such as tackling, may leave children unprepared for physical challenges, dampen sports engagement or fail to yield significant benefits in the long term. This article synthesises emerging evidence, highlights controversies and proposes future directions for policies and research. Ultimately, delaying tackling until the age of 12 years, paired with robust contact skill instruction and progression, may offer a safer path for children’s long-term health and sports participation.

## Key Points

**Table:** 

Developing brain vulnerability: Children under 12 years have unique anatomical, cognitive and neuromuscular traits that heighten SRC risk.
Reducing exposure: Delaying full-contact tackling or body checking lowers total head impacts, potentially preventing acute and cumulative injuries.
Skill development: Postponing contact does not mean neglecting contact skills. Structured, incremental training can help children develop safe and effective techniques without chaotic collisions.
Evidence gaps: High-quality, longitudinal prospective research is still needed to confirm the long-term neurological and psychological benefits of delayed tackling.
Policy implications: Age-based tackling restrictions, combined with robust skill instruction, may be the most pragmatic strategy for safeguarding young athletes’ health.

## Introduction

Sports-related concussions (SRC) in youth sports remain a global public health concern [1, 2]. Team contact sports, such as rugby, American football, ice hockey and Australian rules football, where intentional and forceful contact is integral to gameplay, carry a significantly higher risk of head impacts compared with sports such as soccer (football) or basketball, where contact is incidental and regulated and forceful contacts are penalised [1]. This has led to discussions regarding whether we should delay or ban the full-body contact element (i.e., tackling and bodychecking) for children under a certain age (often proposed as 12 years) [3]. Advocates claim that restricting full contact among preteens can reduce head injury risk, and this would also offer more time for skill development [2–4]. However, some sports organisations and coaches fear that removing contact “softens” the competitive nature of these sports or leaves youth underprepared for the physical demands they will face later [4–6].

Despite the debates, calls for greater caution in exposing young athletes to high-impact collisions have become increasingly pronounced. In youth ice hockey, it has been shown that delaying the introduction of body checking to ages 13–14 years can reduce SRCs without any consequence, i.e., increased SRC rates, once it is introduced [7, 8]. In American football, higher frequency and severity of head impacts were observed in children and adolescents in tackling leagues compared with flag football [7]. Yet, robust longitudinal data tracking early non-contact versus contact exposure and subsequent neurocognitive outcomes remain sparse. This Current Opinion article synthesises recent evidence, acknowledges key controversies and proposes future directions for research and practice on whether we should delay full-body contact in youth contact sports.

## Why the Concern? Children’s Unique Vulnerability

### The Developing Brain

Children’s brains differ substantially from adult brains, not only in size and proportion but also in their composition and developmental trajectory [9, 10]. Compared with adults, children have thinner cranial bones and proportionally larger heads, making them more vulnerable to rotational and linear forces upon impact [10]. Weaker neck musculature further compromises their ability to stabilise the head, thereby increasing the risk of brain movement within the skull when collisions occur [11, 12]. In addition, incomplete myelination means neural pathways are still maturing, potentially rendering them more susceptible to damage from repeated blows [10, 12, 13].

Although definitive evidence on the long-term effects of collisions on youths remains limited, sub-concussive impacts, head impacts that cause pathophysiological changes in brain function without the clinical presentation of concussion, pose a particular concern [12]. Emerging research in adolescents suggests that the earlier age of the first exposure to repetitive sub-concussive injuries, as well as concussions, results in greater loss of caudate volume and greater axonal damage in the frontal lobe [12]. One study found that the younger age of first exposure to American football, before the age of 12 years in particular, was associated with increased odds for impairment in self-reported neuropsychiatric and executive function in 214 former American football players [14]. These low-level hits could affect neural function or structure over time [15–17]. In adult cohorts, repeated head impacts and SRC have been associated with ongoing neurocognitive and mood disturbances, prompting efforts to minimise early exposure to high-impact collisions [15, 18–20].

While these retrospective studies in American football [12, 14], combined with the preliminary findings from youth ice hockey [21–26]*,* suggest potential benefits to delaying contact. The broader body of evidence remains in its infancy. Many conclusions are drawn from observational or retrospective data, with limited original research directly examining the long-term effects of early SRC or repeated sub-concussive impacts. Therefore, high-quality, prospective longitudinal studies are urgently needed to better understand the lasting neurological and psychosocial impacts of early exposure to head impacts.

### Tackling and/or Bracing Techniques and SRC Risk

Mounting evidence from both youth and adult contact sports underlines the critical role that tackling and/or bracing techniques play in SRC risk [7, 8, 27–30]. In collegiate and professional American football, suboptimal tackling approaches, such as leading with the head, have been strongly linked to higher rates of head impacts and SRCs [7]. Similarly, in youth settings, children often lack the ability to consistently tackle using effective head and shoulder placement, making them prone to direct head collisions or dangerous “head-down” tackles [27, 31]. Compounding this, their developing neuromuscular control can hamper their ability to brace effectively or adapt mid-play to an opponent’s movement [32–34].

When players fail to maintain a stable base, align the shoulder correctly or keep the head safely to the side, collision forces transmitted to the brain may escalate [28, 30, 35]. Studies in youth rugby confirm that athletes with better technical proficiency exhibit fewer head injuries [27, 31]. In youth American football, a head-up, vertical-style tackle has also been seen to reduce the number of head accelerations over 10 g (often considered to be high impact) [30]. The concern is that children being introduced to full-contact sports participation without adequate skill development may lead to poor tackling mechanics and increased injury risk. However, it is important to acknowledge that many youth sports systems have significantly advanced their coaching practices in recent years due to the inclusion of coach education programs [36]. National and international governing bodies often mandate annual coach education, with a recent emphasis on SRC awareness and progressive, age-appropriate skill instruction [37]. As such, while some inconsistencies in implementation remain, many youth programs integrate contact exposure with some form of structured, skill-based training [38]. Nonetheless, developing an evidence-based, progressive approach, starting with slow-speed drills, tackle bags and targeted feedback, remains essential for reinforcing safe body positioning and head placement, thereby reducing SRC risk across all levels of competition.

### Neuromuscular Control and Cognitive Immaturity

In addition to skeletal and muscular immaturity, children’s cognitive and motor development may not keep pace with the split-second reactions demanded by contact sports [33, 34, 39]. Mastering tasks such as anticipating tackles, bracing for impact and landing safely involves higher-level perceptual–cognitive and motor skills, which continue to develop throughout childhood [33, 34, 39]. When children lack this foundation, they are more prone to awkward tackles, delayed defensive manoeuvres and difficulty distributing forces evenly upon contact. However, it is worth acknowledging that children who gravitate toward contact sports may have earlier development of coordination and confidence with physical contact. These children may not require the same delay in contact exposure and may feel penalised by a blanket age restriction. Conversely, others may benefit from additional time to build foundational skills. When considering skeletal muscular maturation, it is important to support flexible, skill-based pathways that can accommodate individual differences in physical growth. Policies should reflect this spectrum of physical readiness. Nonetheless, the maturation of the brain must remain a central focus when designing policies to protect young athletes.

## The Argument for Delaying Tackling

### Reducing Overall Exposure to Head Impacts

“Exposure reduction” is a cornerstone of injury prevention [2, 40]. By removing or modifying full-body contact for preteens, we could substantially cut the number of impacts and, by extension, reduce the acute and possible cumulative effects on the developing brain [2]. Considering most SRC mechanisms stem from body contact, it follows that reducing contact should affect incidence rates [31, 41–43]. Evidence from ice hockey supports this approach, leagues that introduced body checking at older ages (13–14 versus 11–12 years) reported lower SRC rates in younger cohorts [7, 8]. Importantly, research indicates that having prior bodychecking or tackling experience does not correlate with fewer SRC once introduced, suggesting no unintended consequences of disallowing full-body contact until later adolescence [7, 44].

### Skill Development in Controlled Settings

A common misconception is that delaying full-body contact means not coaching it at all. In reality, a gradual approach to contact skills, such as body positioning, safe falling and shoulder placement (i.e., when performing a tackle in rugby), ensures these techniques are introduced before athletes face high-intensity collisions [45–47]. This structured skill development begins with low-speed drills using tackle bags and padded surfaces, letting children focus on technique and awareness without immediate live-contact pressure. As proficiency increases, training activities evolve to incorporate greater speed and decision-making, similar to training agility [48]. Therefore, the development of physical contact capabilities is carefully curated through effective coaching practices.

Crucially, by holding off on full-contact until around the age of 12 years, children gain time to master core skills of the game, such as catching, passing, kicking and tactics, free from the added complexity of live-contact pressure [49, 50]. Mastering sport-specific skills first ensures that, when full-contact (i.e., tackling) is introduced, it happens in a context where players are already proficient in key aspects of play. This dual focus on skill-based contact training and fundamental game skills promotes a more holistic athletic development in training contexts that sample key physical and cognitive–emotional aspects of the game [51]. Children who become adept in non-contact facets before facing full-contact scenarios can better maintain effective technique under game conditions, reducing the likelihood of unsafe tackles and improving overall performance [52, 53]. A similar principle has been applied to soccer heading, where it is argued that banning heading during critical skill-development phases may inadvertently foster fear and poor technique, thereby raising future SRC risk once heading is reintroduced [54]. Instead, it is advocated to teach the fundamentals such as ball tracking, body positioning and safe form without ball–head contact early on, ensuring children neither develop harmful heading habits nor perceive heading as unsafe at the age of 10 years but then magically “safe” at the age of 11 years [54].

### Aligning with Physical and Cognitive Maturation

Between the ages of 8 and 12 years, children make significant gains in physical fitness, motor coordination, body awareness and cognitive function, such as reaction time and decision-making [33, 34, 39, 50, 55], and this represents a critical window for taking advantage of children’s heightened neuroplasticity [56]. These developments can enable them to better anticipate plays, gauge distances and control posture under physical stress. For some children, this may promote safer and more ingrained techniques compared with a later introduction. Delaying tackling within competitive games until closer to the age of 12 years capitalises on these advancements, ensuring most children have more refined motor skills, heightened spatial awareness and a stronger neck and torso, all contributing to safer collision management.

Cognitive maturation can also help children process visual cues more effectively so they can position themselves to avoid direct head impacts [39, 57, 58]. An 8-year-old, for instance, may struggle with the rapid judgments required to align the shoulder and brace the neck properly when delivering a tackle in rugby. By 12 years, many can execute these decisions with greater consistency. Aligning physical growth with cognitive readiness can, therefore, reduce the likelihood of severe collisions or repeated head impacts, allowing youth athletes to enter contact scenarios with a firmer grasp of safe technique and the confidence to use it under live conditions.

## The Counterarguments and Controversies

### “Softening” the Sport

Critics may worry that removing or delaying tackling dilutes the competitive and physical essence of contact sports [4, 5]. They argue that physicality is a key component of sports such as Rugby or American football and that “protecting” players too much may lead to fear or inadequate preparation when tackling eventually occurs [4, 5]. Some coaches maintain that the best way to learn safe tackling is through early exposure, allowing children to adapt gradually and develop resilience in real conditions [4, 5].

### Efficacy and Evidence Gaps

While several observational and retrospective studies suggest reduced SRC rates with limited contact, high-quality, long-term data remain limited [7, 8]. Rigorous prospective trials tracking children who avoid full-body contact until 12 years versus those who engage in full-body contact earlier would offer more convincing cause–effect evidence. Furthermore, confounding variables, such as coaching expertise, athlete genetics or league competition levels, further complicate direct comparisons. It is also not uncommon for youth leagues to change multiple factors at once (e.g. smaller teams, modified fields, no contact), making it hard to isolate the effect of delayed tackling.

### Implementation Feasibility

Sports differ in how easily they can modify rules to exclude or delay tackling. Rugby and American football rely heavily on the tackle for ball possession changes yet could implement flag or touch formats. Ice hockey separates “bodychecking” from “non–bodychecking” leagues, and Australian football could theoretically adopt a contact model closer to Gaelic football (shoulder-to-shoulder only, similar to what is allowed in some under-9-years competitions). For smaller sports organisations with volunteer coaches, enforcing contact bans or systematic skill training may pose logistical challenges. Additionally, families who value competitive, hard-hitting play may resist a shift toward reduced-contact programs.

## Existing Models and Recent Data

### Non-tackle or Flag Variants

In Rugby and American football, touch or flag adaptations offer a means to limit collision exposure in younger players while preserving the strategic elements of contact sports. These varieties emphasise ball-handling, evasion and tactical positioning without the risk of full-contact tackles. This progressive skill development allows children to transition gradually to the tackle variants as they mature physically and cognitively. Overall, non-tackle or flag variants serve as a valuable “stepping stone” for youth athletes, laying a foundation of spatial awareness, coordination and game intelligence that can be further built upon once contact is introduced. Numerous studies [59–61] have reported significantly higher rates of head impacts in the full-contact variants of the same sport compared with their non-contact counterparts, such as flag or touch formats. However, the rules can fundamentally differ, which may not always adequately prepare players for the full-contact variety.

Modified tackle versions such as USA Football’s Rookie Tackle [62] and TackleBar [63], which modify field size, reduce team rosters and curtail contact for children under 12 years, offer sport-specific “bridge” examples of incorporating full-body contact. While not an outright no-tackle policy, these modifications significantly decrease collision frequency and intensity. Early findings indicate fewer high-impact collisions and more repetitions of fundamental skills, such as passing and evasion, during practices and games, enhancing body awareness and technique before heavier-contact play [64].

### Weight Categories

Some American football and Rugby competitions employ weight-based or size-based categories to reduce mismatches in junior competitions, aiming to lower the injury risk posed by disparities in body mass and strength [65, 66]. By grouping children of similar size, leagues hope to mitigate “overpowered” tackles where heavier players collide with much lighter opponents. Although this system does not entirely eliminate concerns about head impacts, it can create a more developmentally appropriate environment.

One study found that youth athletes in weight-restricted categories experienced fewer SRC, likely due to more balanced physical matchups [65]. Still, body size alone is not the only factor influencing SRC risk, as smaller players may still sustain head impacts if they lack proper technique or neuromuscular control. Moreover, weight categories do not address potential skill or cognitive readiness gaps, both of which also affect collision outcomes. Despite limitations, this approach represents a concerted effort to minimise size disparities in contact team sports and aligns with broader calls for safer youth sports practices.

### Body-Checking Policies in Ice Hockey

Ice hockey offers one of the most thoroughly studied examples of modifying contact rules among youth. Historically, body checking began at the ages of 11–12 years, but some leagues have raised this threshold to 13–14 years to reduce injuries. Multiple studies [21–26] have observed a significant drop in injury rates among younger (under 13 years) cohorts when body checking was delayed. Further, prior experience with bodychecking in games was not linked to increased SRC rates in leagues that allowed checking, suggesting no unintended negative effects from delaying its introduction [67, 68]. This suggests that “later is safer” for exposing children to high-impact collisions. Although improved helmet technology, coaching certifications and heightened SRC awareness may partially explain these findings, the overarching principle is that reducing contact exposure at younger ages can lead to fewer serious injuries.

Nonetheless, the relationship between delayed body checking and SRC reduction is not always straightforward, with a number of factors influencing SRC risk (e.g. coaching quality, athlete size, league structure). Critics contend that postponing body checking may inadvertently delay skill acquisition, potentially causing anxiety or inadequate technique when adolescents eventually face full-contact sports participation [4–6]. Even so, the majority of evidence underscores that a developmentally aligned contact introduction can positively shape youth injury profiles, reinforcing similarly cautious strategies in other contact sports.

## Potential Pathways Forward

### Gradual, Skill-Based Contact Training

Contemporary skill development and coaching frameworks, such as the Phases of Skill Training (PoST) [69] approach and the Specificity, Progression, Overload, Reversibility and Tedium (SPORT) [70] conceptual model, underscore the need for periodised progression when learning complex motor skills, even though there is no universal consensus on precisely how to structure this process in youth sports. Translating these ideas into contact sports means introducing fundamental body-contact skills in controlled, low-intensity environments before transitioning into competitive, chaotic settings. By aligning contact-skill training with children’s physical and cognitive readiness, coaches can reduce SRC risk while still promoting robust skill development through tailored learning environments.

A graded approach to implementing full contact might include:

- Progressive contact drills: Build proficiency in initiating and receiving tackles, starting at lower intensities and gradually increasing speed.
- Neck and trunk strength training: Reinforce protective musculature to stabilise the head during collisions.
- Safe falling and bracing techniques: Teach youth athletes to land without excessive head or neck movement, incorporating progressively more dynamic drills.
- Perceptual–cognitive and vision training: Improve athletes’ perceptual–cognitive, motor processing and visual performance, equipping them to anticipate and react to sudden contact scenarios.

**Fig. 1:**
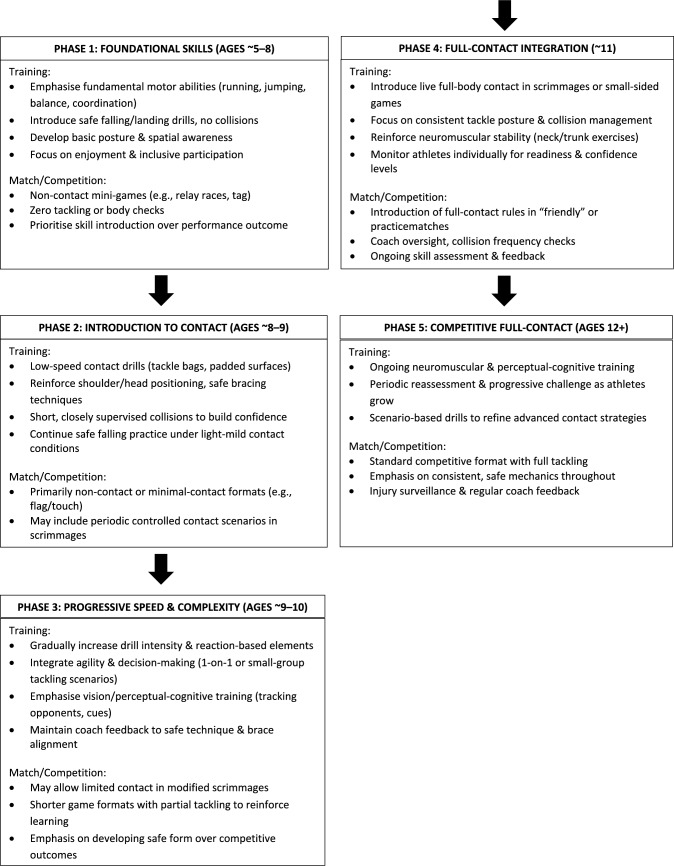
Flow diagram of an example phased contact progression that ensures that children acquire fundamental skills and develop safe contact techniques before facing fully competitive, high-speed collisions, thereby reducing the risk of sports-related concussions and other injuries

Taken together, these components offer a structured, skill-based roadmap that aligns with developmental science, enabling children to acquire the necessary techniques for safe contact play without prematurely exposing them to high-stakes collisions. Building on skill development frameworks such as PoST [69] and SPORT [70], Fig. 1 depicts an example of a phased approach from foundational non-contact skills to full-body contact and was designed with existing developmental programs such as the TackleReady program [38] in mind.

### Unified Age-Based Guidelines

A “no-full-contact-before-12-years-of-age” policy would standardise rules across youth leagues, removing ambiguity. Programs such as World Rugby’s “Activate” and federation-level mandates already cover age-appropriate skill modules [71], but no universal requirement exists on precisely when to introduce full-body contact. Adopting consistent guidelines could also benefit multi-sport athletes or those who switch codes during their developmental years, ensuring they do not encounter mismatched policies (e.g. a sport that introduces contact at 10 years versus one that waits until 12 years), which can lead to confusion and uneven skill acquisition. Players crossing over from a non-contact environment into one that already permits contact, i.e., tackling, would otherwise face the dual challenge of mastering a new sport while simultaneously coping with potentially higher-intensity physical contact. By unifying the age of introduction for contact skills, all children, regardless of the code they play, would receive adequate, progressive training before stepping into full-contact scenarios within competitive games. Uniform guidelines could, therefore, reduce confusion and ensure every child receives consistent, developmentally aligned instruction, not just those in elite or well-funded setups.

### Research Directions: Tracking Long-Term Outcomes

Future prospective, longitudinal studies should compare:

1. Early contact (contact before the age of 12 years) versus delayed contact (no full contact until 12 years), measuring SRC incidence, sub-concussive hits, neurocognitive outcomes and skill acquisition.
2. The effectiveness of various training protocols (e.g. neck strengthening, progressive contact drills, cognitive/vision training) in preventing head injuries once contact is introduced.
3. Psychological and developmental impacts: Does delaying tackling foster more anxiety or less confidence, or do children adapt if they have undergone structured, progressive skill training?

### Stakeholder Engagement and Policy Collaboration

Achieving meaningful changes depends on multi-stakeholder buy-in. Governing bodies, youth leagues, coaches, parents and medical professionals must work together to define feasible guidelines. Clear communication about the rationale for delaying tackling, and the distinction between skill-based contact drills versus unregulated collisions, can alleviate concerns that youth sports are being “watered down.” Continuous monitoring and feedback loops are essential to refine rules as new data emerge.

## Conclusion

Should we ban or delay full-body contact i.e., tackling, in youth contact sports before the age of 12 years? Current evidence suggests that a cautious approach, particularly one that reduces contact exposure in the preteen years, may support long-term neurological safety without impairing skill development or engagement. Younger athletes’ developing brains, less mature neuromuscular control and limited cognitive readiness make them more vulnerable to both acute and repetitive head impacts. We argue that delaying full-body contact can provide time for structured, sport-specific skill development in the short-term, providing a platform for safer and more sustained participation in contact sports long-term.

However, regardless of the specific timing or structure of contact introduction, one consistent factor underpins successful outcomes, the quality of coaching. Coaches who have the training, time and support to deliver evidence-based, individualised and developmentally appropriate instruction are essential to safe and effective player development. Indeed, it is often the coach who determines whether children thrive, stay safe and remain engaged in sport through effective instruction and a shared passion for the sport.

Nonetheless, epidemiological, physiological and developmental findings converge on a rationale for minimising full-contact in the preteen years. With structured coaching, skill-based drills and protective measures (i.e., neck strengthening and perceptual–cognitive training), children can learn how to impart and receive contact safely once they are better prepared both physically and psychosocially. For the millions of children worldwide who love these sports, balancing fun, skill and safety will be key to retaining participation and promoting long-term brain health.
